# The effects of DNA supercoiling on G-quadruplex formation

**DOI:** 10.1093/nar/gkx856

**Published:** 2017-09-28

**Authors:** Doreen A.T. Sekibo, Keith R. Fox

**Affiliations:** Biological Sciences, Life Sciences Building 85, University of Southampton, Southampton SO17 1BJ, UK

## Abstract

Guanine-rich DNAs can fold into four-stranded structures that contain stacks of G-quartets. Bioinformatics studies have revealed that G-rich sequences with the potential to adopt these structures are unevenly distributed throughout genomes, and are especially found in gene promoter regions. With the exception of the single-stranded telomeric DNA, all genomic G-rich sequences will always be present along with their C-rich complements, and quadruplex formation will be in competition with the corresponding Watson–Crick duplex. Quadruplex formation must therefore first require local dissociation (melting) of the duplex strands. Since negative supercoiling is known to facilitate the formation of alternative DNA structures, we have investigated G-quadruplex formation within negatively supercoiled DNA plasmids. Plasmids containing multiple copies of (G_3_T)_*n*_ and (G_3_T_4_)_*n*_ repeats, were probed with dimethylsulphate, potassium permanganate and S1 nuclease. While dimethylsulphate footprinting revealed some evidence for G-quadruplex formation in (G_3_T)_*n*_ sequences, this was not affected by supercoiling, and permanganate failed to detect exposed thymines in the loop regions. (G_3_T_4_)_*n*_ sequences were not protected from DMS and showed no reaction with permanganate. Similarly, both S1 nuclease and 2D gel electrophoresis of DNA topoisomers did not detect any supercoil-dependent structural transitions. These results suggest that negative supercoiling alone is not sufficient to drive G-quadruplex formation.

## INTRODUCTION

Guanine-rich DNA sequences can fold into four-stranded structures that consist of stacks of G-quartets (1–5). Sequences with the potential to adopt this structure are common throughout the human genome (6,7), though they are unevenly distributed and are frequently found in the proximal promoter region of genes, particularly those involved in growth-control and in several oncogenes (8–11).

The formation of G-quadruplexes in G-rich single stranded oligonucleotides *in vitro* is facile, requiring only the presence of monovalent cations such as potassium. However, the question of their formation *in vivo* has been a matter of considerable debate (12) especially since, with the exception of the 3′-ends of telomeres, all genomic G-rich sequences are normally associated with their complementary C-rich strands. Several *in vitro* studies have shown that, in linear fragments under physiological conditions, G-rich sequences usually form duplexes rather than quadruplexes (13–19). However, using a quadruplex-specific antibody, G-quadruplexes were first visualised in the macronuclei of ciliate *Stylonychia lemnae* (20). Biffi *et al.* (21) subsequently described the use of an antibody (BG4) with high selectivity and low nanomolar affinity for G-quadruplex DNA to visualise G-quadruplex formation in human cells. This study showed that the number of G-quadruplexes increased during S phase, indicating that their structural formation was dependent on DNA replication, a point at which the DNA strands are transiently separated at the replication fork, allowing the single strands to fold into secondary structures. This antibody has also been used to demonstrate elevated levels of G-quadruplex formation in stomach and liver cancer tissues (22). A similar study by Henderson *et al.* (23,24) described the development and characterisation of another monoclonal antibody (1H6), which indicated the formation of quadruplex structures throughout the genome *in vivo*. Quadruplexes have also been directly mapped in purified genomic DNA (25).

Strand separation, which must precede quadruplex formation from duplex DNA, is known to be facilitated by negative supercoiling and many studies have demonstrated the formation of non-B DNA structures, such as triplexes (H-DNA), Z-DNA and cruciforms, within plasmids under negative superhelical stress (26–32). However, there have, been few studies on the effect of supercoiling on G-quadruplex formation. Early studies (33) showed that long repeats of the *Tetrahymena* telomeric sequence (GGGGTT)_*n*_.(AACCCC)_*n*_ were sensitive to S1 nuclease under conditions of high negative supercoiling and were preferentially cleaved on the C-rich strand. Chemical probing, along with two-dimensional electrophoresis of plasmids containing similar G_4_T_2_ repeats showed the formation of non-B DNA structures, but only under conditions of very low pH (4.4) with no transition observed at pH 5.0 (34). However, neither of these early studies attributed these change to quadruplex formation. Other early studies with repeats of the human telomeric sequence (GGGTTA)_*n*_ showed the formation of non-B structures in supercoiled plasmids, but only at very low pH (4.0) and with long repeats (n>8) (35,36). More recently, a chemical footprinting study on the NHE III1 polypurine/polypyrimidine tract of the *c-myc* promoter (37) revealed that, in the presence of 100 mM KCl, negative superhelicity induced the formation of a G-quadruplex structure in the four guanine tracts at the 5′-end. Interestingly, this study also revealed i-motif formation in the C-rich complement, even though this usually only forms at low pH. *In vitro* footprinting of the cloned VEGF G-quadruplex-forming sequence also demonstrated the formation of quadruplex structures in supercoiled DNA, with and without the presence of stabilising ligands (38). Conversely, a plasmid clone containing the quadruplex forming region of the *bcl-2* gene (39) was unable to form a G-quadruplex under negative supercoiling without the addition of short peptide nucleic acids (PNAs) that bound to the complementary C-rich strand, displacing the G-strand, thereby making it available for quadruplex formation. A further study showed that invasion by the PNA was dependent on formation of the G-quadruplex structure (40). DNA gyrase has also been shown to facilitate the formation of G-quadruplexes in circular DNA duplex (41). Using magnetic tweezers, Selvam *et al.* (42), suggested that G-quadruplex formation correlated with DNA melting, increasing to 23% at σ < −0.05. Zhang *et al.* (43) also used chemical probing *in vitro* to show that negative supercoiling induced by downstream transcriptional events facilitated the formation of G-quadruplex structure(s) (43). These data demonstrated that G-quadruplex formation in dsDNA could be triggered thousands of bases pairs away. They also suggested that G-quadruplex-forming sequences may serve as sensors for remote DNA tracking activity in response to the propagation of mechanical torsion in DNA double-helix.

In this paper, we have investigated the effects of DNA supercoiling on the formation of model intramolecular G-quadruplexes that contain simple G_3_ repeats separated by short (T) or long (T_4_) loops. Quadruplexes with short loops generally adopt a parallel topology and form more stable structures than those with longer loops, which adopt an antiparallel topology (44–49). (G_3_T)_*n*_ forms one of the most stable intramolecular quadruplexes (14), and it should therefore be most likely to show a supercoil-dependent transition from duplex to quadruplex conformation. We therefore cloned synthetic oligonucleotides containing quadruplex-forming sequences with short (G_3_T)_*n*_ and long (G_3_T_4_)_*n*_ loops into pUC plasmid vectors and used chemical and enzymic probes to examine quadruplex formation in supercoiled and linear plasmids.

## MATERIALS AND METHODS

### Oligonucleotides and enzymes

The oligonucleotides used for cloning were synthesised by ATDBio (Southampton) on an Applied Biosystems 394 DNA/RNA synthesiser. Oligonucleotides were prepared with a 5′-phosphate group to facilitate ligation. [α-^32^P]-dATP was purchased from PerkinElmer. Accugel and Ureagel were obtained from National Diagnostics. All enzymes were purchased from Promega or New England Biolabs except for AMV reverse transcriptase, which was purchased from Sigma-Aldrich.

### Plasmid clones of quadruplex forming sequences

The G-rich sequences listed in Table 1 were cloned into the BamHI site of pUC19. These sequences were chosen to represent quadruplex forming sequences with short [(G_3_T)_*n*_] or long [(G_3_T_4_)_*n*_] loops. Since many G-rich biological sequences contain more than four G_3_-tracts, we prepared plasmids with four or five G_3_ repeats, and each of these was obtained as a monomeric or dimeric insert. The ligated plasmids were transformed into *Escherichia coli* TG2 and plated onto agar plates containing 0.02% X-gal, 1 mM IPTG and 100 μg/ml carbenicillin. White colonies were picked from these and sequenced by Eurofins MWG Operon. The clones obtained were oriented such that the G-rich strand was visualized when sequencing with the Universal reverse primer, except for [(G_3_T)_4_]_2_ and (G_3_T)_5_ which were obtained in the opposite orientation. For the chemical probing experiments the G-rich strand was therefore visualized by labelling the 3′-end of the EcoRI site, except for [(G_3_T)_4_]_2_ and (G_3_T)_5_ which were labelled at the 3′-end of the HindIII site. Plasmid samples were purified using a QIAprep spin miniprep kit (Qiagen) according to the manufacturer's instructions.

**Table 1.** Sequences of inserts cloned into the BamHI site of pUC19

	Sequence
(G_3_T)_4_	5′-GATCGGGTGGGTGGGTGGG
	CCCACCCACCCACCCTAG-5′
(G_3_T)_5_	5′-GATCGGGTGGGTGGGTGGGTGGG
	CCCACCCACCCACCCACCCCTAG-5′
[(G_3_T)_4_]_2_	5′-GATCGGGTGGGTGGGTGGGGATCGGGTGGGTGGGTGGG
	CCCACCCACCCACCCCTAGCCCACCCACCCACCCTAG-5′
[(G_3_T)_5_]_2_	5′-GATCGGGTGGGTGGGTGGGTGGGGATCGGGTGGGTGGGTGGGTGGG
	CCCACCCACCCACCCACCCCTAGCCCACCCACCCACCCACCCCTAG-5′
(G_3_T_4_)_4_	5′-GATCGGGTTTTGGGTTTTGGGTTTTGGG
	CCCAAAACCCAAAACCCAAAACCCCTAG-5′
(G_3_T_4_)_5_	5′-GATCGGGTTTTGGGTTTTGGGTTTTGGGTTTTGGG
	CCCAAAACCCAAAACCCAAAACCCAAAACCCCTAG-5′
[(G_3_T_4_)_4_]_2_	5′-GATCGGGTTTTGGGTTTTGGGTTTTGGGTTTTGATCGGGTTTTGGGTTTTGGGTTTTGGG
	CCCAAAACCCAAAACCCAAAACCCAAAACTAGCCCAAAACCCAAAACCCAAAACCCCTAG-5′
[(G_3_T_4_)_5_]_2_	5′-GATCGGGTTTTGGGTTTTGGGTTTTGGGTTTTGGGGATCGGGTTTTGGGTTTTGGGTTTTGGGTTTTGGG
	CCCAAAACCCAAAACCCAAAACCCAAAACCCCTAGCCCAAAACCCAAAACCCAAAACCCAAAACCCCTAG-5′

Plasmid clones were oriented such that the G-rich strand was visualised when sequencing with the Universal reverse primer, except for [(G_3_T)_4_]_2_ and (G_3_T)_5_ which were obtained in the opposite orientation.

### Reaction with dimethylsulphate

For the reaction with supercoiled DNA, 150–200 ng plasmid was incubated overnight in 50 μl of 10 mM Tris–HCl pH 7.5 containing 100 mM KCl. Dimethylsulphate (0.5 μl of a 1% v/v solution) was then added and the reaction was stopped after 1 min by addition of 1 μl β-mercaptoethanol. The reaction mixture was precipitated with ethanol, redissolved and cleaved with EcoRI and PstI (except for plasmids containing the clones of [(G_3_T)_4_]_2_ and (G_3_T)_5_ which were cut with HindIII and SacI). The modified DNA was then labelled at the 3′-end with [α-^32^P]dATP using AMV reverse transcriptase and the fragments were separated on an 8% non-denaturing polyacrylamide gel. The radioabelled bands of interest were eluted into 10 mM Tris–HCl pH 7.5 containing 0.1 mM EDTA, precipitated and heated in 10% (v/v) piperidine at 95°C for 30 min. The piperidine was removed by lyophilisation, followed by several washes with water, each followed by lyophilisation. The sample was finally redissolved in formamide containing 10 mM EDTA and bromophenol blue, heated at 100°C for 3 min before loading onto a denaturing polyacrylamide gel. The reaction with linear DNA was performed in a similar way by first cutting the plasmid (150–200 ng) with EcoRI and PstI (or HindIII and SacI), before reacting with dimethylsulphate, and radiolabelling with [α-^32^P]dATP as described for the supercoiled DNA.

### Reaction with permanganate

150–200 ng plasmid DNA was incubated overnight in 50 μl of 10 mM Tris-HCl pH 7.5 containing 100 mM KCl. 0.5 μl of 100 mM KMnO_4_ was then added and the reaction was stopped after 20 min by adding 1 μl of β-mercaptoethanol. All the other steps were then as described for dimethylsulphate.

### S1 nuclease

Plasmid DNA (dissolved in 10 mM Tris-HCl pH 7.4 containing 100 mM KCl) was diluted in 20 μl S1 nuclease buffer (50 mM M sodium acetate pH 4.5 containing 280 mM NaCl and 4.5mM ZnSO_4_). This was digested with eight units S1 nuclease (Promega) for 30 min. DNA was precipitated with ethanol, redissolved and digested with (8–12 units) of restriction enzyme ScaI. The reaction with linear DNA was determined by reversing the order of addition of ScaI and S1 nuclease.

### Polyacrylamide gel electrophoresis

The products of the cleavage reactions were separated on 8% denaturing polyacrylamide gels containing 8M urea. After electrophoresis, the gels were fixed in 10% (v/v) acetic acid, dried and subjected to autoradiography using a phosphorimager screen (Kodak), which was scanned with a Typhoon phosphorimager.

### Circular dichroism

CD measurements were performed using a Jasco J-720 spectropolarimeter. Oligonucleotides were diluted to 5 μM in 10 mM Tris–HCl, pH 7.5, containing 150 mM KCl. The samples were first heated to 95°C for 10 min and annealed by slow cooling overnight to room temperature. Spectra were recorded between 220 and 320 nm in a 1 mm path length quartz cuvette and were averaged over 16 scans at a scan rate of 100 nm.min^−1^ with a response time of 2 s and 1 nm bandwidth. A spectrum of the Tris buffer was used as the buffer baseline, and the spectra were normalized to have zero ellipticity at 320 nm.

### 2D-electrophoresis with DNA topoisomers

2D gel electrophoresis was performed as described by Bowater *et al.*, (50). Supercoiled plasmid samples (∼300 ng/μl) were incubated with varying concentrations of ethidium bromide (0–2.0 μg/ml) and treated with 1 μl of wheat germ topoisomerase I for 1 hr at 37°C to generate plasmids with varying superhelical density. Samples containing an even distribution of topoisomers were prepared by combining these in appropriate ratios as judged from the intensity of the various bands on 1D agarose gels. A sample of the mixed topoisomer distribution (2.5 μg DNA) was loaded onto a 1% agarose gel (20 cm × 20 cm) and subjected to electrophoresis in 1× TBE buffer for 16–18 h at 3.0 V/cm. The gel was then soaked for 8–10 h in TBE buffer containing chloroquine diphosphate (2–10 μg/ml) in the dark. The gel was rotated by 90° and run in the second dimension in 1× TBE buffer containing the same concentration of chloroquine, for 16–18 h at 3.0 V/cm. After electrophoresis, the gel was stained with GelRed™ (1 μl/100 ml) in water overnight. The gel was then visualized and photographed using a Syngene, G-Box transilluminator. From these 2D gels we estimate that the native superhelical density (σ) of these plasmids was about –0.075.

## RESULTS

### Circular dichroism

Before studying the properties of these G-rich sequences within supercoiled plasmids, we first used circular dichroism to assess quadruplex formation by the synthetic oligonucleotides alone, either as G-rich single strands or in combination with their C-rich complements. The results are shown in Supplementary Figures S1 (monomers) and S2 (dimers). Several previous studies have shown that quadruplex-forming sequences with single base loops typically adopt a parallel topology with a positive peak around 260 nm, while those with longer loops form antiparallel structures with a peak around 295 nm (51–53). This is confirmed by the results shown in Supplementary Figures S1 and S2 for the single-stranded G-rich oligonucleotides (solid lines), which display peaks around 260 nm for sequences with single-T loops and 290 nm for those with T_4_-loops. As expected, addition of the complementary C-rich strands causes dramatic changes in the CD spectra of the sequences with T_4_-loops (dashed lines), as the two strands form a Watson–Crick duplex. However, this is not seen with the sequences that contain short loops, for which there are only small changes in the CD spectra on adding the C-rich complements, which retain the characteristic peaks around 265 nm. This is consistent with our previous results, from fluorescence melting experiments (14), which also demonstrated that sequences containing repeats of (G_3_T) retained a quadruplex structure in the presence of an excess of the complementary C-rich strand, while most other quadruplex-forming sequences formed a duplex in preference to the quadruplex.

### Dimethylsulphate protection

**Figure 1.**
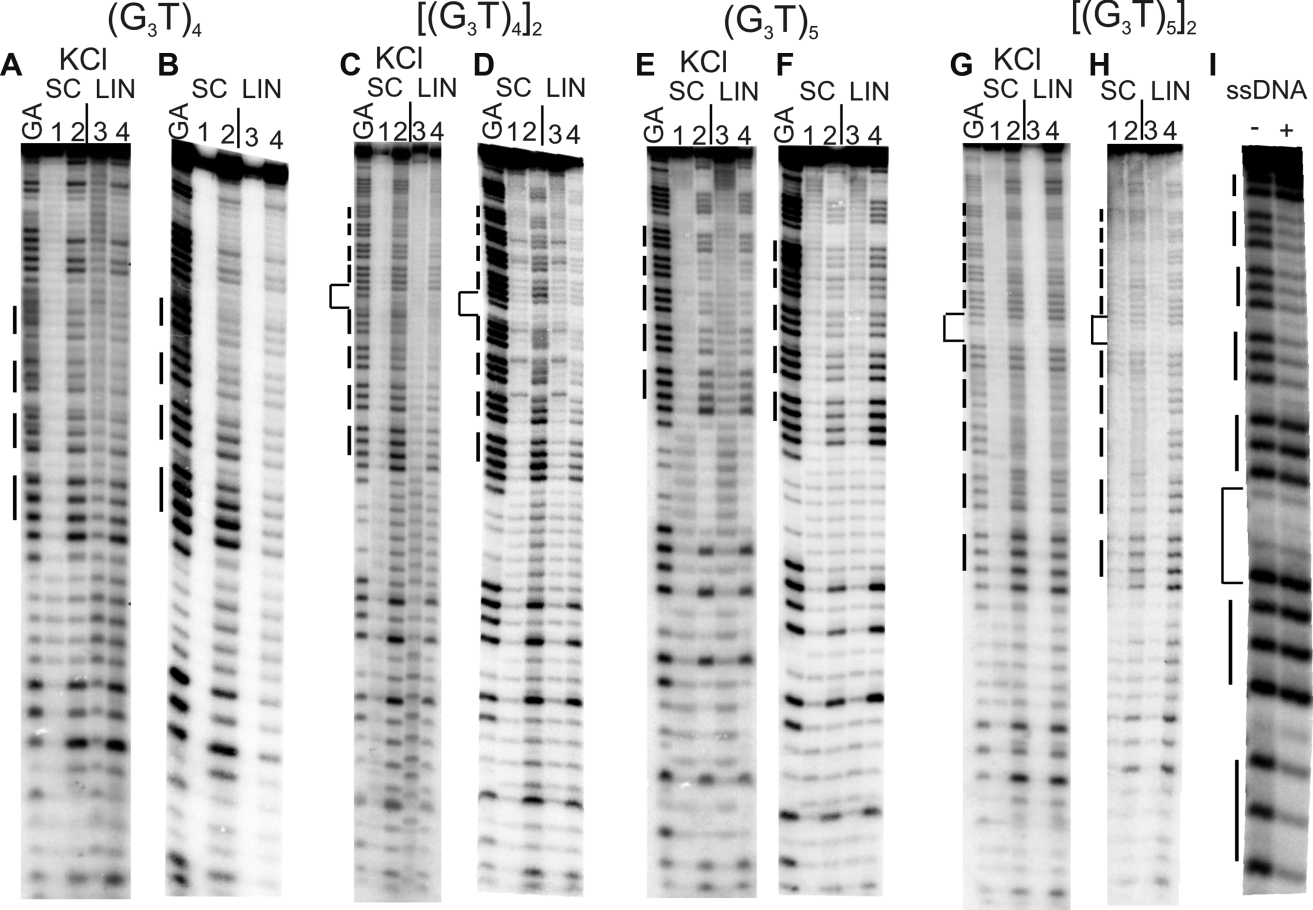
Reaction of the (G_3_T)_*n*_-containing plasmid inserts with dimethylsulphate (DMS). Supercoiled (SC) and linearised (LIN) plasmids were modified with DMS, in the presence (A, C, E, G) or absence (B, D, F, G) of 100 mM KCl. Lanes labelled 1 and 3 are untreated DNA, while lanes 2 and 4 correspond to reaction with dimethylsulphate. The final panel (I) shows the reaction of DMS with the single stranded oligonucleotide [(G_3_T)_5_]_2_ (labelled at the 5′-end) in the absence (-) and presence of 100 mM KCl. The plasmids were incubated in buffer overnight before adding DMS. Tracks labelled GA correspond to Maxam-Gilbert markers specific for purines. The locations of the G_3_ blocks are indicated by the filled boxes. For the dimeric inserts the central GATC is indicated by a bracket.

**Figure 2.**
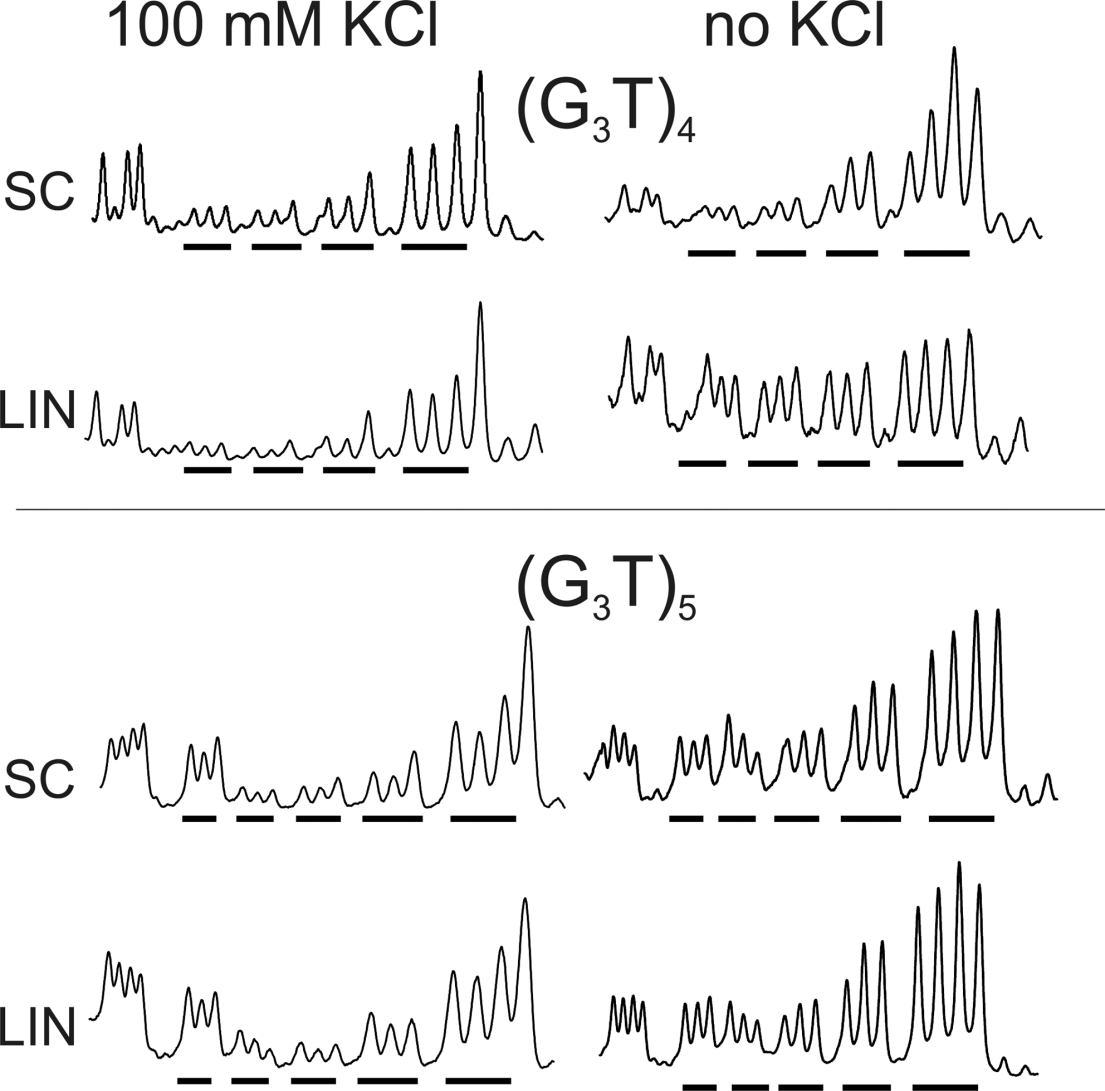
Densitometric scans of the reaction of dimethylsulphate with the (G_3_T)_4_ and (G_3_T)_5_ plasmid inserts in the presence (left) and absence (right) of 100 mM KCl. The sequences run from 5′-3′- left to right and the locations of the G_3_ tracts are indicated by the filled bars. SC, supercoiled DNA; LIN linear DNA.

**Figure 3.**
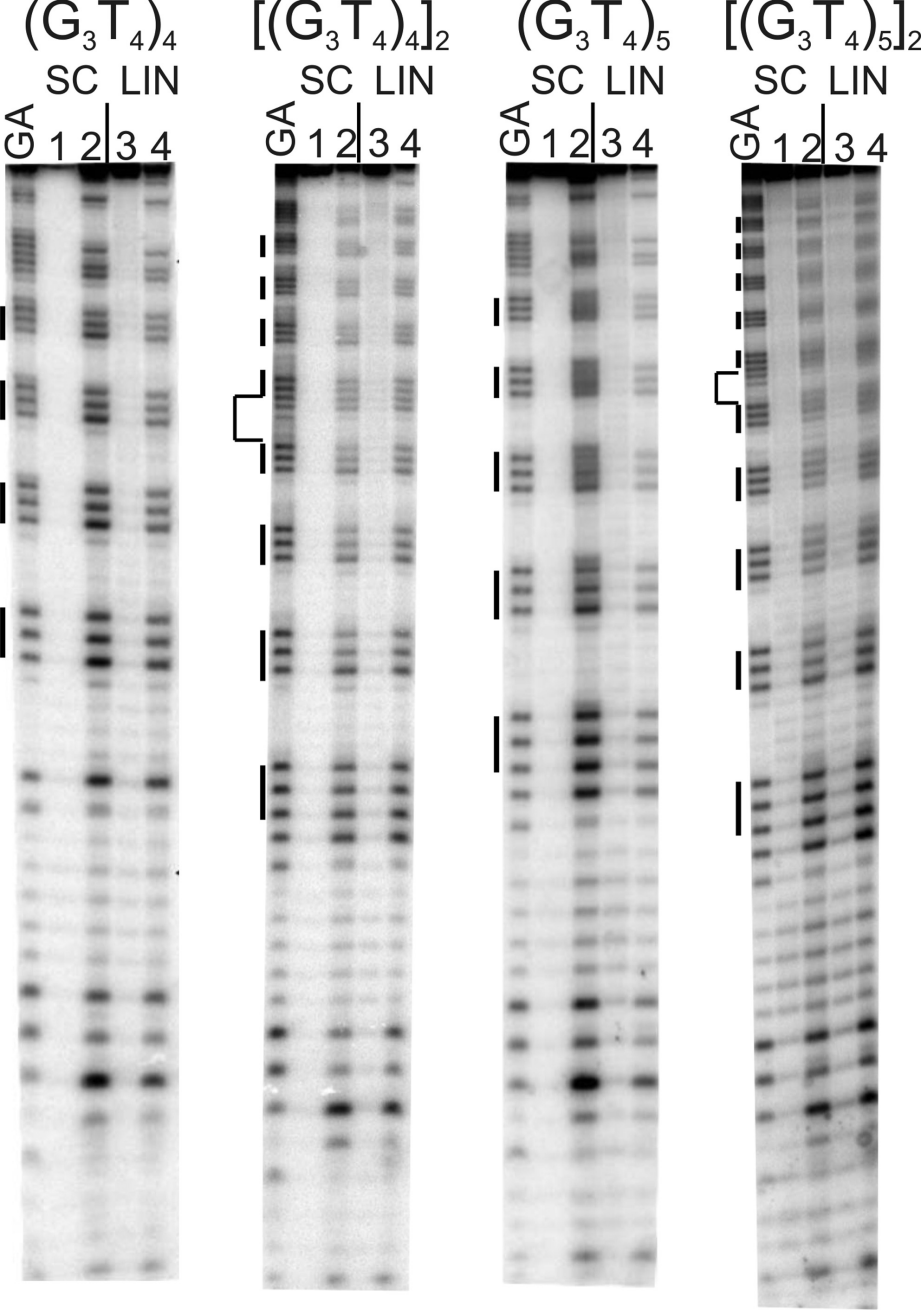
Reaction of the (G_3_T_4_)_*n*_-containing plasmid inserts with dimethylsulphate (DMS). *S*upercoiled (SC) and linearised (LIN) plasmids were modified with DMS in the presence of 100 mM KCl. Lanes labelled 1 and 3 are untreated DNA, while lanes 2 and 4 correspond to reaction with DMS. The plasmids were incubated in buffer overnight before adding DMS. Tracks labelled GA correspond to Maxam-Gilbert markers specific for purines. The locations of the G_3_ blocks are indicated by the filled boxes. For the dimeric inserts (second and fourth panels) the central GATC is indicated by a bracket.

**Figure 4.**
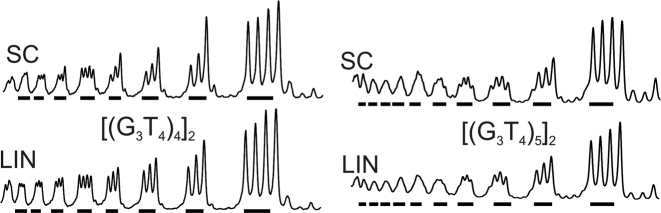
Densitometric scans of the reaction of dimethylsulphate with the (G_3_T_4_)_4_ and (G_3_T_4_)_5_ plasmid inserts in the presence (left) and absence (right) of 100 mM KCl. The sequence runs from 5′-3′- left to right and the locations of the G_3_ tracts are indicated by the filled bars. SC, supercoiled DNA; LIN linear DNA.

Dimethylsulphate (DMS) reacts with N7 of guanine leading to strand cleavage after subsequent treatment with piperidine. This nitrogen is occluded in G-quartets and DMS protection has therefore been widely used to assess G-quadruplex formation. In the following experiments, we assessed the reactivity of guanines within several cloned synthetic G-tracts to DMS, comparing the behaviour of supercoiled and linear DNA fragments. These experiments were performed in the presence of potassium ions (100 mM KCl) in order to facilitate quadruplex formation, as well as in potassium-free buffers. The results are presented in Figures 1 and 3, along with representative densitometer traces of the cleavage patterns in Figures 2 and 4. Densitometer profiles for the other sequences are included in the Supplementary material (Supplementary Figure S3 and S4).

#### Single T-loops

Intramolecular quadruplexes containing short loops are generally very stable and fold into a parallel structure (46,47). Figure 1A and B shows the reaction of DMS with G-rich sequences in which four successive G_3_ tracts are separated by single T residues [(G_3_T)_4_]. Figure 1A shows the results in the presence of potassium, while Figure 1B presents similar experiments in a potassium-free buffer. Figure 1C and D shows the results of similar experiments with a dimer of this sequence in which the repeats are separated by GATC. In the presence of potassium (Figure 1A and C) the G_3_-tracts in these DNA fragments display reduced reactivity to DMS, compared with other guanines in the rest of the sequence. G_3_ tracts at the 5′-(upper) end of each insert are less reactive than those at the 3′-(lower) end. These variations in intensity are more readily seen in the densitometric scans shown in Figure 2 (monomeric inserts) and Supplementary Figure S3 (dimeric inserts). The bands for the uppermost (5′) G_3_ tracts are about 20% the intensity of guanines in the rest of the sequence, with greater reaction at the other two G-tracts, which is still lower than reaction with the Gs in the rest of the fragment. Similar patterns are also produced in both repeats of the dimeric insert, with the greatest reduction in DMS reaction in the G_3_-tracts at the 5′-(upper) end of each repeat. These patterns are indicative of successful G-quadruplex formation. However, in each case a similar pattern of modification is evident in both the linear and supercoiled samples. These data appear to provide good evidence for the formation of G-quadruplexes in these G_3_T inserts, but surprisingly this does not seem to depend on negative supercoiling and the protections are apparent in the linear fragments. Surprisingly, experiments performed in the absence of potassium (Figure 1B and D) also show similar attenuations in DMS reaction with supercoiled DNA fragments, though this is much less pronounced in the linear fragments.

Since many biological sequences that have the potential to adopt G-quadruplexes contain more than four G_3_-tracts, we performed similar experiments with constructs that contain five successive G_3_ tracts, each separated by single thymine residues. The results are presented in Figure 1E–H for the monomeric and dimeric inserts of (G_3_T)_5_ and densitometric traces of these are shown in Figure 2 and Supplementary Figure S3. In the presence of 100 mM potassium, guanines in the centre of these tracts show attenuated reaction with DMS in both the supercoiled and linear substrates. The protection is greater for the central three G_3_ tracts than for the first and fifth, and is especially pronounced for the second and third tracts (counting from the upper (5′)-end). A sequence containing five tandem G_3_-tracts can adopt several conformations, utilizing either the first four or last four guanines in the folded quadruplex (i.e. excluding the first or fifth G_3_ tract from the quadruplex). The DMS methylation pattern suggests that the DNA sample contains a mixture of both these structures. As noted for the sequence with four G_3_ repeats the cleavage patterns in the presence of potassium are similar for the linear and supercoiled substrates. Attenuated reaction is also seen in absence of potassium for the supercoiled DNA, though the cleavage pattern is more even in the potassium-free buffer.

The final panel of Figure 1 (Figure 1I) shows the reaction of DMS with the single stranded oligonucleotide [(G_3_T)_5_]_2_ (labelled at the 5′-end with ^32^P) in the absence and presence of 10 mM KCl. As seen with the plasmid insert of this sequence, the second, third and fourth G_3_ repeats of the upper (3′) part of this dimer show attenuated reaction with DMS in the presence, but not the absence, of 100 mM KCl. Only the two G_3_ tracts closest to the 3′-end of the lower (5′) part of this dimer are resolved on the gel, but again the tract corresponding to the fourth G_3_ is protected, while the fifth is not.

#### T4-loops

Intramolecular quadruplexes with longer loops are generally less stable than those with short loops and they fold more slowly into antiparallel structures (46,47,54). The first two panels of Figure 3 show the results of DMS protection experiments for DNA fragments containing four G_3_ tracts that are separated by T_4_-loops, in the presence of 100 mM potassium. Densitometric scans of these data are shown in Figure 4 (monomers) and Supplementary Figure S4 (dimers). In these experiments, every guanine in the G-rich regions has the same intensity as those in the surrounding sequences, in both the linear and supercoiled DNA samples. A similar result is seen in the absence of potassium (not shown). Similar results are seen with the dimeric inserts of this sequence (Figure 3, third and fourth panels), in which the intensity of every guanine is very similar. These patterns suggest that this sequence does not form a G-quadruplex in either supercoiled or linear fragments, even in the presence of 100 mM potassium.

The results of DMS modification with plasmids that contain five G_3_ tracts separated by T_4_-loops, in the presence of potassium are shown in the third and fourth panels of Figure 3 for the monomeric and dimeric inserts respectively, and densitometric scans of these data are shown in Figure 4 and Supplementary Figure S4. Once again, all the guanines showed the same reactivity to DMS in both linear and supercoiled substrates, in both the presence and absence of KCl (not shown). These patterns suggest that no G-quadruplex structures have formed in these sequences with T_4_ loops in either the linear or the supercoiled fragments.

### Reaction with permanganate

Permanganate reacts with exposed thymines, by out-of-plane attack on the 5,6 double bond (55). Fully base paired thymines in B-DNA are unreactive to this probe, but it is has been widely used to detect the presence of single-stranded regions, unpaired thymines or distortions in the DNA helix (56–58). We therefore anticipated that it would be able to react with thymines in the loops of the quadruplexes.

**Figure 5.**
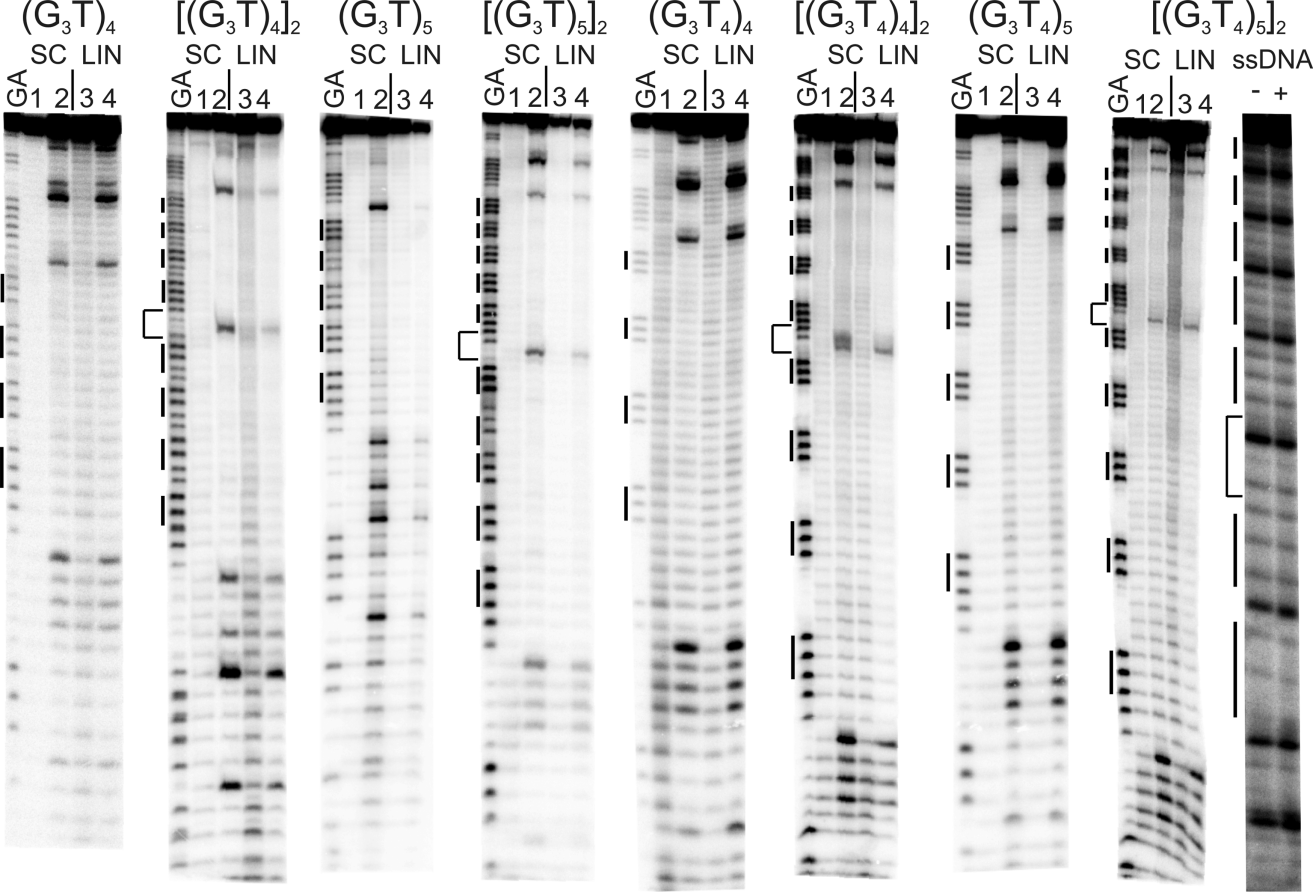
Reaction of the (G_3_T)_*n*_- and (G_3_T_4_)_*n*_-containing plasmid inserts with potassium permanganate. *S*upercoiled (SC) and linearized (LIN) plasmids were modified with permanganate in the presence of 100 mM KCl. Lanes labelled 1 and 3 are untreated DNA, while lanes 2 and 4 correspond to reaction with permanganate. The plasmids were incubated in buffer overnight before adding permanganate. The final panel shows the reaction of permanganate with the single stranded oligonucleotide [(G_3_T)_5_]_2_ (labelled at the 5′-end) in the absence (–) and presence (+) of 100 mM KCl. Tracks labelled GA correspond to Maxam-Gilbert markers specific for purines. The locations of the G_3_ blocks are indicated by the filled boxes. For the dimeric inserts the central GATC is indicated by a bracket.

Figure 5 shows the reaction of permanganate with all the DNA fragments containing the G_3_-tracts, in the presence of 100 mM KCl. In each case, the reaction with supercoiled DNA (lane 2) is compared with linear DNA (lane 4). For each sequence, bands can be seen at thymines that flank the G-rich repeat sequence in both the supercoiled and linear DNA samples, though no reaction is evident at any thymine residues in the proposed loop regions. For the fragments labelled at the EcoRI site reactive thymines can be seen above the inserts at 5′-CTAGAGGAT and below at 3′ (GATC), while for those labelled at the HindIII site [[(G_3_T)_4_]_2_ and (G_3_T)_5_] reactive thymines are evident at GATC (above) and GATCCTCTAGAGTC (below). In addition, Ts at the centre of the dimeric inserts (GATC) are also sensitive to modification with this probe. These Ts are more accessible than those within the G-tracts, suggesting that the Ts within the potential quadruplex forming sequences are less exposed and so are protected from reaction with permanganate. Although the absolute intensity of the bands for [(G_3_T)_4_]_2_ and (G_3_T)_5_] (both labelled at the EcoRI site) is weaker for the supercoiled than the linear DNA, the relative cleavage pattern is the same for both DNA samples, and no specific thymines are hypersensitive to permanganate.

The final panel of Figure 5 shows the reaction of permanganate with the single stranded oligonucleotide [(G_3_T)_5_]_2_ (labelled at the 5′-end with ^32^P). In this case the thymines within each G_3_T repeat show the same reactivity to permanganate in the absence and presence of 100 mm KCl, confirming that quadruplex formation itself does not occlude these residues. The reactivity of the thymine in the central GATC is also the same as those in the G_3_-tracts and contrasts with the results with the supercoiled samples.

### Cleavage with S1 nuclease

**Figure 6.**
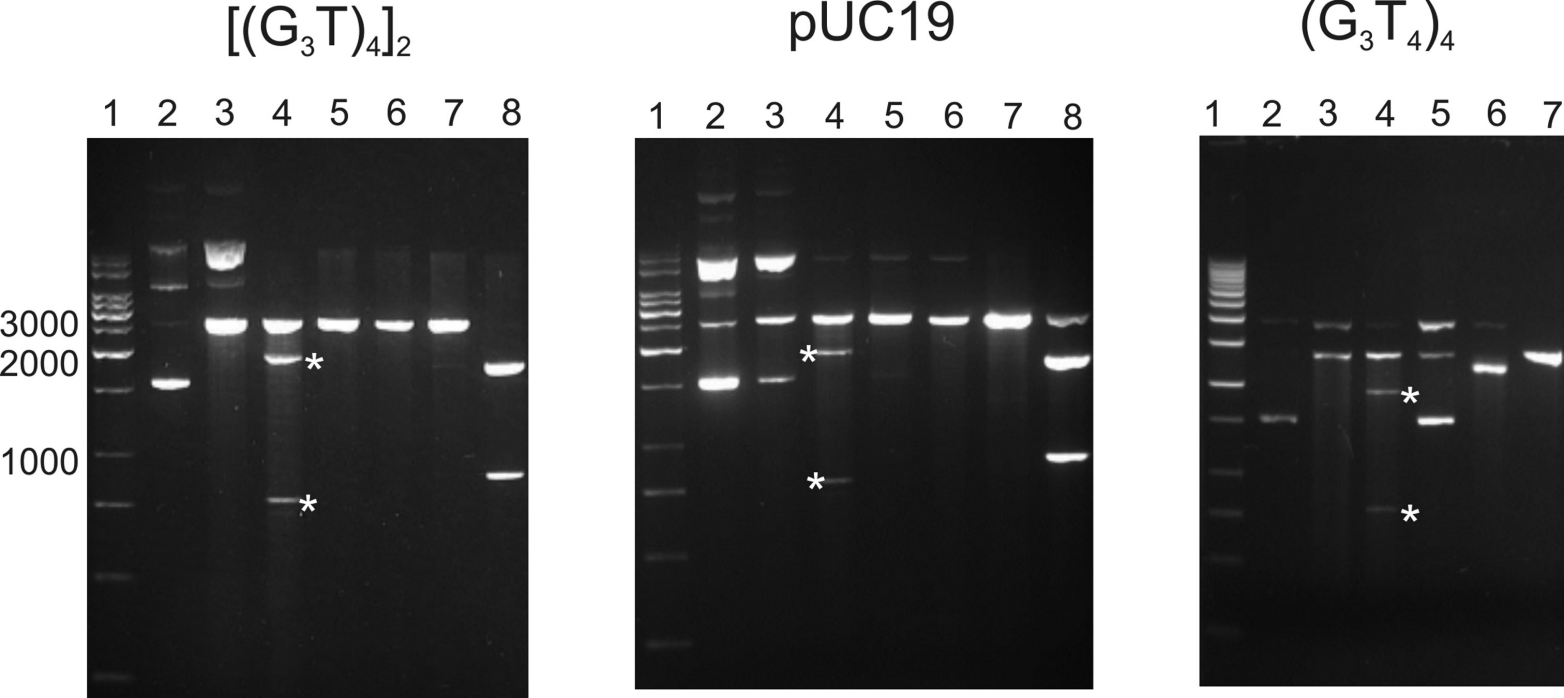
Mapping of S1 nuclease-sensitive sites in pUC19 and plasmids containing [(G_3_T)_4_]_2_ and (G_3_T_4_)_4_ and inserts. Lane 1, DNA size marker; lane 2, native supercoiled DNA; lane 3, cleavage with S1 nuclease; lane 4, digestion with S1 nuclease followed by ScaI; lane 5 digestion with ScaI; lane 6, digestion with *Sca*1 followed by S1 nuclease; lane 7, digestion with EcoRI; lane 8, digestion with EcoRI and ScaI. The products of S1 nuclease followed by ScaI digestion are indicated by the asterisks.

S1 nuclease cuts single-stranded regions of DNA, and has previously been used to probe for other non-B DNA structures, such as H-DNA and cruciforms. The formation of quadruplexes within these plasmids should generate single stranded regions in the loops, at the quadruplex-duplex junctions and within the displaced C-rich strands. We therefore used this enzyme to supplement the results of the chemical probing experiments. Each supercoiled plasmid was digested with S1 nuclease, followed by ScaI in order to locate the position of any S1 cleavage site(s). Reversing the order of addition of these enzymes will linearise the plasmids before adding the S1 and so will remove any supercoil-dependent regions of single stranded DNA. The results of these experiments are shown in Figure 6. The left hand panel of Figure 6 shows S1 mapping of the plasmid containing the inserts [(G_3_T)_4_]_2_. S1 digestion, followed by ScaI cleavage generates two fragments (indicated by asterisks) with sizes of approximately 1900 and 760 bp (lane 3), which are not evident when the order of these two enzymes is reversed (lane 6). This suggests that S1 has cut at a specific location in the plasmid. However these fragment sizes do not correspond those expected for G-quadruplex formation (1760 and 925 bp), which should be the same as those produced in the double digest by EcoRI and ScaI (lane 8). This is explained by the observation that the same pattern is seen with the parent pUC19 plasmid (Figure 6, middle panel), which also contains a supercoil-dependent S1 nuclease sensitive site that is eliminated when the plasmid is linearised. pUC19, contains a palindromic 11 bp inverted repeat sequence around position 1385, which has previously been shown to form a weak cruciform structure under superhelical stress (31,59,60), for which digestion with S1 and ScaI should produce DNA fragments of 1890 and 790 bp. The cleavage pattern is therefore indicative of native cruciform formation, rather than the presence of a quadruplex. A similar effect is seen with the plasmid containing (G_3_T_4_)_4_, which shows the banding pattern for cruciform rather than quadruplex formation. This lack of supercoil dependent quadruplex formation is consistent with the results of the chemical probing experiments, though these low pH conditions are different from those used for chemical probing, which might promote i-motif formation in the complementary C-rich strand.

### 2D-gel electrophoresis

Supplementary Figure S5 shows the 2D gel electrophoresis pattern of pUC19 topoisomers alongside those for plasmids containing the inserts [(G_3_T)_4_]_2_ and [(G_3_T_4_)_4_]_2_, performed in the presence of 1 mM KCl. For all these plasmids the mobility of each topoisomer increases periodically with increasing superhelical density to form a smooth curve. No discontinuous transitions were seen in the profile of either of the plasmids containing G-rich inserts, indicating that no supercoil-dependent conformational changes had occurred.

### A sequence with an inverted G-rich repeat

During the cloning of the G-rich plasmids an unusual mutant was isolated that contained an imperfect combination of three (G_3_T_4_)_*n*_ sequences, the last of which is inverted. This plasmid could adopt several isomeric quadruplex structures as well as a cruciform based around the repeat. It was also subjected to the same chemical modifications and S1 nuclease analyses as the other plasmids, and the combined results are presented in Supplementary Figure S6.

As seen with all the other plasmids containing G-tracts separated by T_4_ loops, the reaction with dimethylsulphate (not shown) failed to show any protection within the G-tracts nor any supercoil-dependent effects. The first panel of Supplementary Figure S6 shows the results of reaction with permanganate. Once again, there is no reaction with thymines in any of the potential T4-loop regions. However, hyperreactive bases can be seen with the supercoiled DNA in the (GATCC) region between the G_3_ and C_3_ tracts, with especially strong cleavage at the central thymine. The second panel shows the results of S1 cleavage of this plasmid, in which subsequent digestion with ScaI (lane 4) generated four fragments, indicated by asterisks, with sizes of approximately 2000, 1800, 900 and 760 bp. This indicates the presence of specific supercoil-dependent single strands, which are not evident when the plasmid was linearised before treating with S1. The DNA cleavage pattern of this sample suggests that the supercoiled plasmid contains two regions that are sensitive to S1 cleavage, one of which is near the G-rich insert. However, this may not reflect quadruplex formation as this sequence contains a 25 bp C_3_A_4_-sequence that is an inverted repeat of the preceding block of G_3_T_4_, from which it is separated by the sequence GATC, which includes the hyperreactive T. This sequence can therefore form either a G-quadruplex or a cruciform (as illustrated in the final panel of Supplementary Figure S6) and the data are consistent with cruciform, rather than quadruplex formation.

## DISCUSSION

The results presented in this paper suggest that under these conditions superhelical density alone is not sufficient to drive G-quadruplex formation within DNA plasmids. Repeats of four or five G_3_ tracts, with either single Ts or T_4_ linkers showed no evidence for quadruplex formation in experiments with permanganate, S1 nuclease cleavage or 2-dimensional gel electrophoresis. Dimethylsulphate modification showed some protections within the G_3_ tracts, though this did not depend on DNA supercoiling, since similar protections were observed with linear DNA fragments. Of course, this does not imply that quadruplexes cannot occur *in vivo*, but it does suggest that they are unlikely to be static structures existing without the influence of other factors. It should, however, be noted that extrapolation of results from small plasmids to large chromosomes is not always valid (61), and higher levels of supercoiling might yet promote quadruplex extrusion.

At first sight, this result is surprising, since other non-B structures can be generated under the influence of superhelical stress, including H-DNA, Z-DNA and cruciforms. However, the kinetic pathway from duplex to intramolecular quadruplex is likely to be very different. Firstly, quadruplexes contain G-rich sequences and their assembly will necessarily involve local unpairing of duplex regions with high GC-content. The kinetic barrier to quadruplex formation will therefore be much higher. Secondly, cruciforms and H-DNA can form via a ‘bubble’ or ‘zipper’ mechanism, in which only a small region of the duplex unfolds in the initial stages, forming a local non-B DNA structure, which is subsequently propagated into neighbouring sequences. In contrast, assembly of the first G-quartet will require extrusion of a relatively long single-stranded region, even if the process involves a transient G-triplex (62–64). This is similar to the sequence-dependent kinetic mechanisms for cruciform formation under different ionic conditions (65,66). S-type extrusion occurs via a small central bubble, which is then propagated in both directions, while C-type extrusion requires a large bubble that involves initial unpairing of a large section of the duplex. This slow kinetic process may explain the unusual pattern that is seen in the 2D gels with the sequence that contains the G-rich inverted repeat (Supplementary Figure S6), which shows the presence of non-interconverting forms, within this GC-rich cruciform.

The inability to form intramolecular quadruplexes under superhelical stress is consistent with previous observations with the *Tetrahymena* telomeric repeat, which only formed non-B structures at extremely low pH (34). Although these results were not interpreted in terms of quadruplex formation, postulating instead the presence of C^+^.C and A^+^.A base pairs, these would also be consistent with i-motif formation by the displaced C-rich strand, which is susceptible to cleavage by S1 nuclease (33). This is also consistent with experiments on plasmids containing repeats of the human telomeric sequence (GGGTTA)_*n*_, which only showed the formation of non-B conformations at very low pH (35,36). However these results contrast with two more recent studies that suggested supercoil-dependent quadruplex formation in the c-myc and VEGF promoters (37,38). We cannot explain this difference, which may be related to the different sequences, plasmids or ionic conditions that were used, or maybe affected by the unusual stability of the c-myc and VEGF i-motifs, which are formed under these physiological conditions (37). The lack of quadruplex formation with sequences containing the T_4_ loops is also consistent with previous studies showing that only short loops induce genome instability by G-quadruplex-forming minisatellites (67). It should also be noted that the pUC plasmids used in the present study, also contained a weak cruciform sequence, which is part of the pMB1 origin of replication. In the S1 nuclease experiments cleavage at the cruciform will relieve the torsional stress that is required to induce the G-quadruplex structure (and *vice versa*; quadruplex formation will relieve superhelical stress and prevent cruciform formation). If there is any competition between the cruciform and the quadruplex, it is clear that the weak cruciform is favoured under these conditions. We note that this weak cruciform-forming sequence is present in many of the plasmids that have previously been used for probing the formation of Z-DNA and H-DNA (such as pRW790), confirming that supercoil-dependent extrusion of quadruplex DNA is much less favoured than formation of these other non-B DNA structures. AT-rich palindromes will be more likely to form competing cruciforms, though this weak cruciform in pUC19 is only 44% AT.

In contrast, the DMS footprinting results suggest that quadruplex formation may occur in the (G_3_T)_*n*_ repeats. Attenuated DMS cleavage is evident for *n* = 4 and 5 in both monomeric and dimeric inserts. The results for the (G_3_T)_5_ repeats are especially informative as the central G_3_-tracts are more strongly protected than outer ones, as would be expected, since quadruplexes could form using four G_3_-tracts at either the 5′- or 3′-end of the (G_3_)_5_ repeat. However, the same result is seen with the linear DNA fragments, demonstrating that the formation of any non-B DNA structures is independent of supercoiling. This is consistent with the CD results, which showed no change in the spectrum on adding the C-rich complement, in contrast to the oligonucleotides with the G_3_T_4_ repeats, which showed the usual duplex formation with the C-strand. This is also similar to our previous fluorescence melting data, which showed that this quadruplex is extremely stable and does not readily form a duplex (14).

None of the inserts showed any reaction of permanganate with the Ts in the loops; in fact, these residues are less reactive than Ts within the other parts of the fragments. This is surprising for the sequences with single T loops, for which DMS showed some evidence for (supercoil-independent) quadruplex formation. It is possible that this base is oriented with the C5-C6 bond facing towards the quadruplex core and therefore inaccessible to permanganate, even on quadruplex formation. However experiments with the single stranded oligonucleotide (final panel in Figure 5) showed that these thymines are susceptible to permanganate modification on quadruplex formation, as expected. It is known that, oligo-T tracts in duplex DNA are refractory to reaction with permanganate, as a consequence of the rigid structure of dA.dT tracts (68). This may explain the general lack of reactivity of the T_4_ loops, though the previous study suggested that the 3′-Ts are not protected from reaction, in contrast to the present results. The low reactivity of the loop thymines in both the supercoiled and linear fragments is therefore a property of the local structure of this duplex, rather than quadruplex formation.

These results suggest that these synthetic simple sequences do not form quadruplexes under superhelical stress, though (G_3_T)_n_ repeats show some evidence for the presence of intramolecular quadruplexes, which is not supercoil-dependent. Nonetheless, several other studies have shown strong evidence for the presence of quadruplexes *in vivo*. It seems reasonable to suppose that other processes that locally separate the DNA strands, such as replication, transcription or protein binding might act together with DNA supercoiling to promote quadruplex formation.

## Supplementary Material

Supplementary Data
