# Supplementary material for: The effects of DNA supercoiling on G-quadruplex formation
Source: Nucleic Acids Res. 2017 Sep 28;45(21):12069–79. doi: 10.1093/nar/gkx856 (PMC5716088; doi:10.1093/nar/gkx856)
Supplement: Supplementary Data [file gkx856_supp.pdf]

The effects of DNA supercoiling on G-quadruplex formation

Doreen A.T. Sekibo and Keith R. Fox*

Biological Sciences, Life Sciences Building 85, University of Southampton, Southampton SO17 1BJ, UK

SUPPLEMENTARY MATERIAL

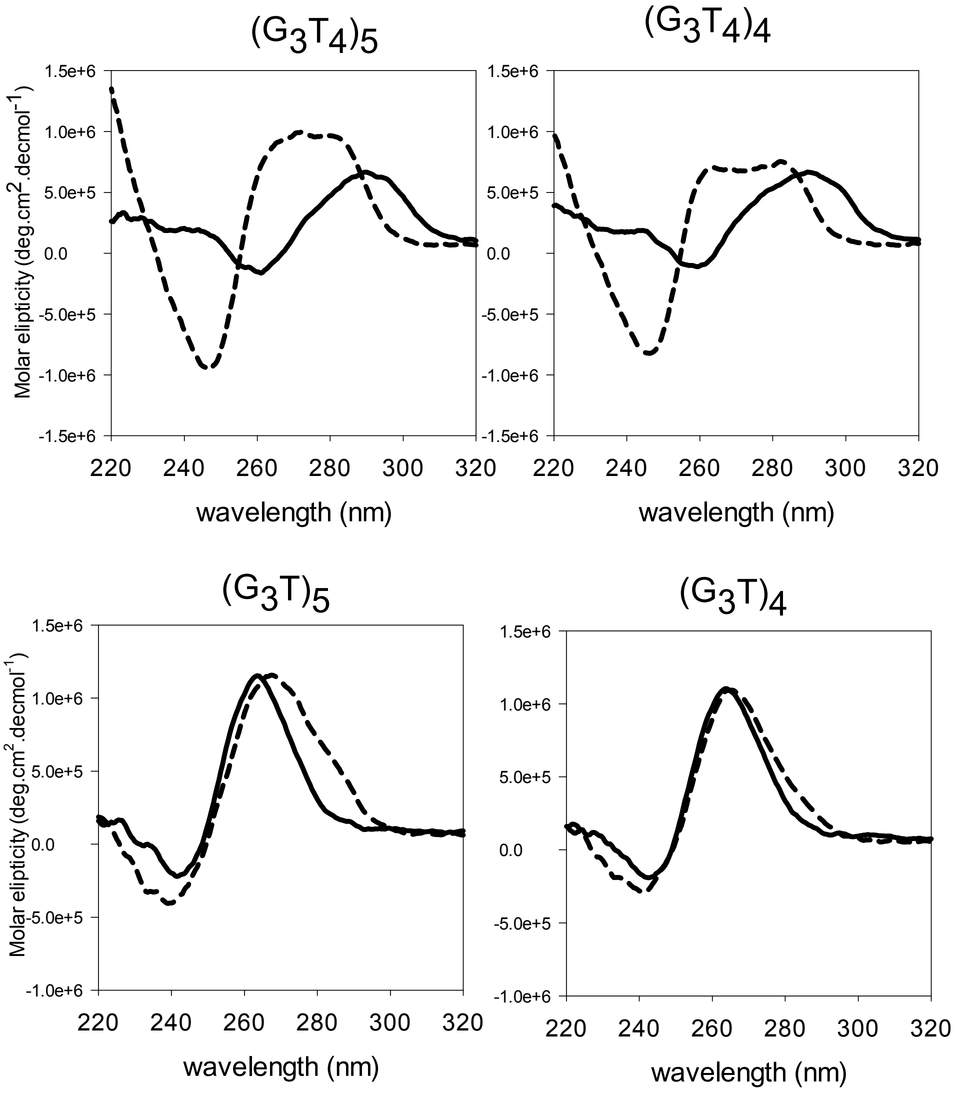

Figure S1. CD spectra of the quadruplex-forming oligonucleotides containing $(G_3T_4)_4$, $(G_3T_4)_5$, $(G_3T)_4$ and $(G_3T)_5$ in the absence (solid line) and presence (dashed line) of the complementary C-rich strands. In each case the oligonucleotide concentration was (5 μM), dissolved in 10 mM Tris-HCl pH7.4 containing 150 mM KCl.

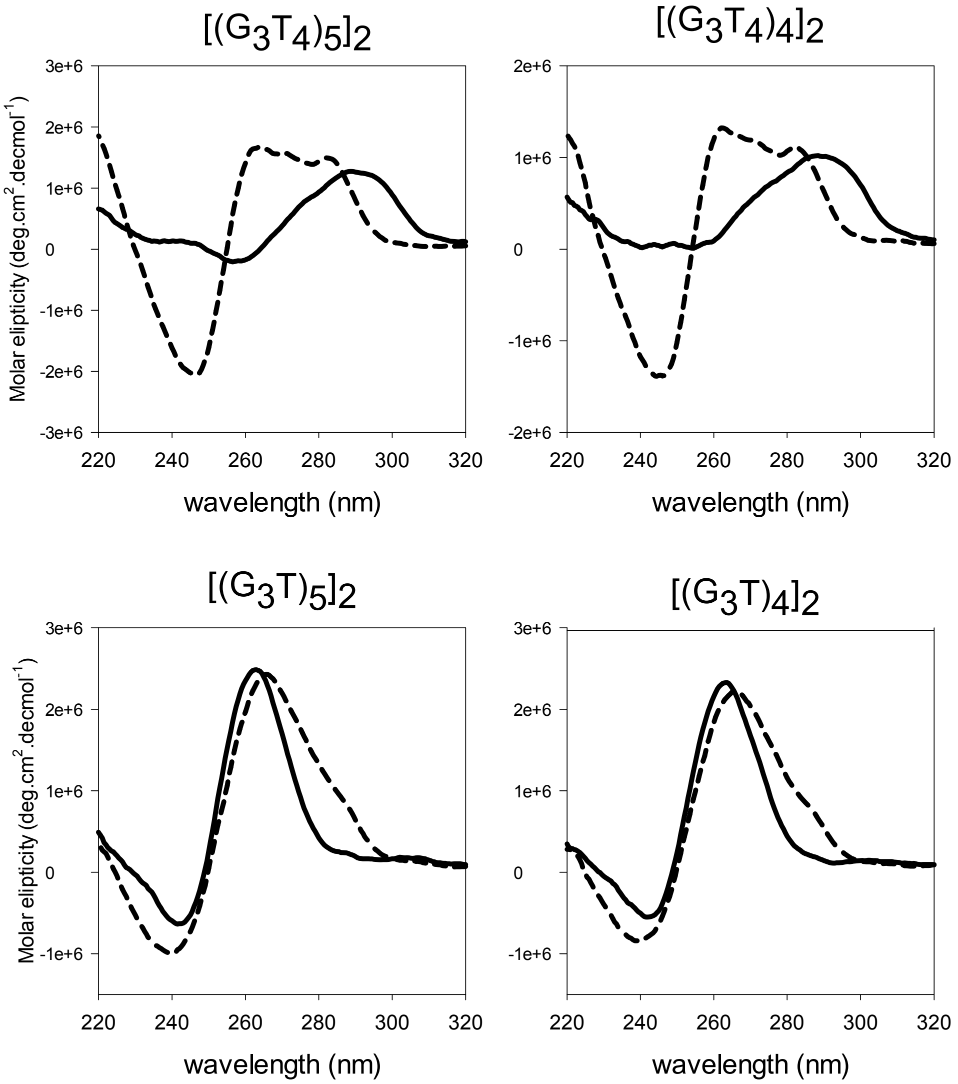

Figure S2. CD spectra of the quadruplex-forming oligonucleotides containing dimers of $(G_3T_4)_4$, $(G_3T_4)_5$, $(G_3T)_4$ and $(G_3T)_5$ in the absence (solid line) and presence (dashed line) of the complementary C-rich strands. In each case the oligonucleotide concentration was (5 μM), dissolved in 10 mM Tris-HCl pH7.4 containing 150 mM KCl.

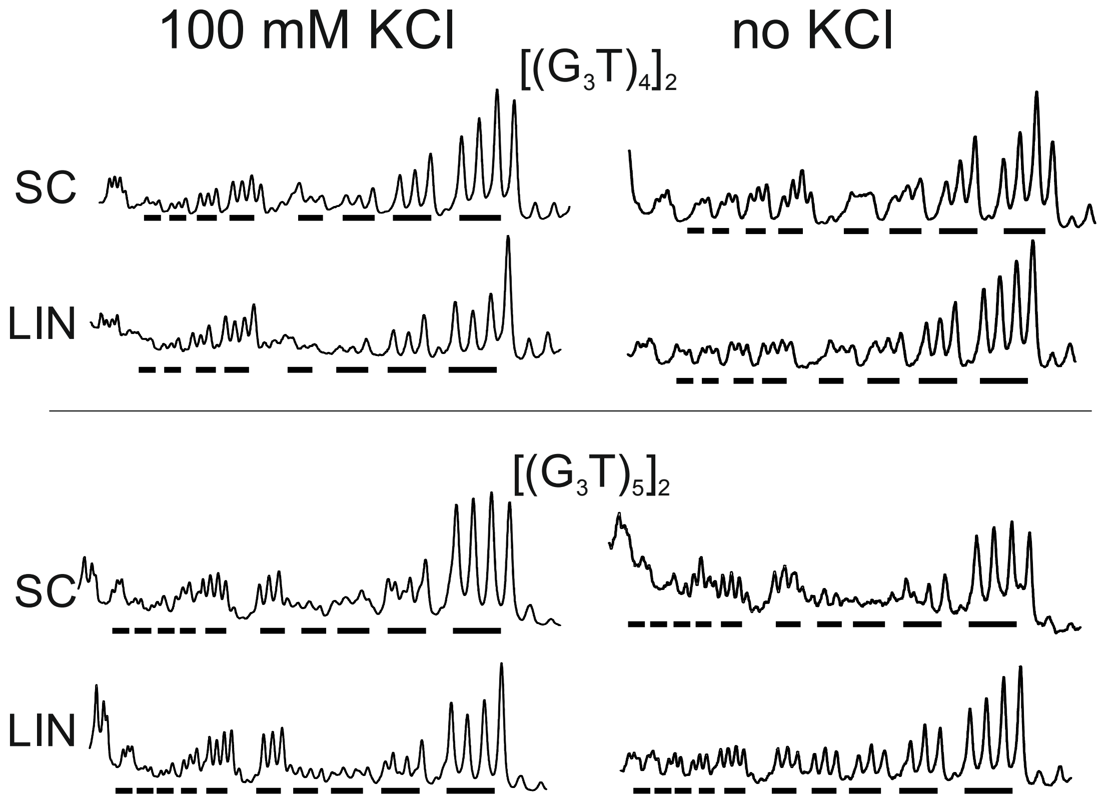

Figure S3. Densitometric scans of the reaction of dimethylsulphate with the dimeric plasmid inserts of $(G_3T)_4$ and $(G_3T)_5$ in the presence (left) and absence (right) of 100 mM KCl. The sequence runs from 5'-3'- left to right and the locations of the G_3 tracts are indicated by the filled bars. SC, supercoiled DNA; LIN linear DNA.

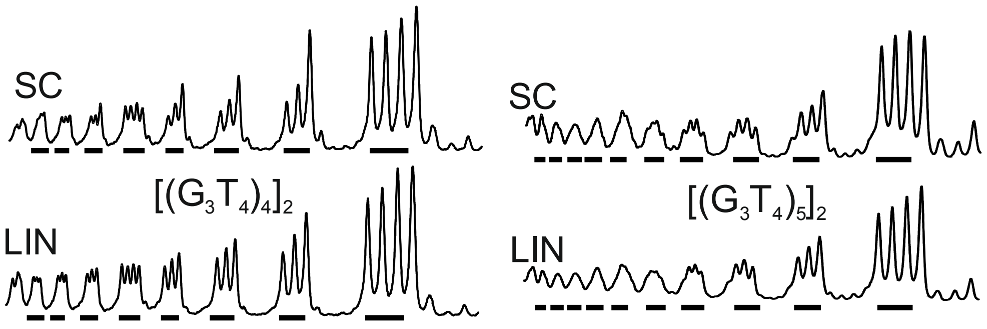

Figure S4. Densitometric scans of the reaction of dimethylsulphate with the dimeric plasmid inserts of $(G_3T_4)_4$ and $(G_3T_4)_5$ in the presence of 100 mM KCl. The sequence runs from 5'-3'- left to right and the locations of the G_3 tracts are indicated by the filled bars. SC, supercoiled DNA; LIN linear DNA.

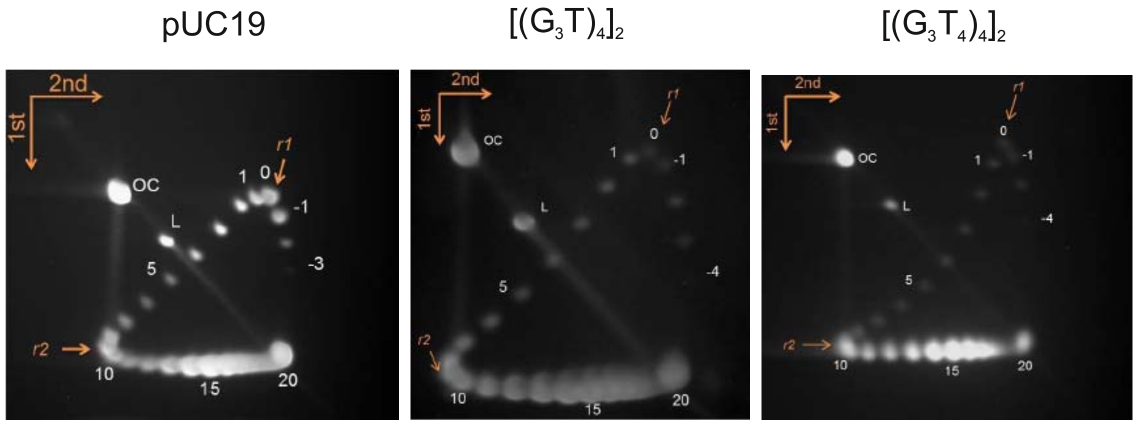

Figure S5. Two-dimensional electrophoresis of mixtures of topoisomers of plasmid pUC19 and the clones containing dimeric inserts of $(G_3T)_4$ and $(G_3T_4)_4$. 1% agarose gels were run in 1xTBE buffer supplemented with 1 mM KCl. After running in the first dimension, the gel was placed in a dark container and soaked in TBE supplemented with 2 μg/ml chloroquine for about 7 hours. Electrophoresis in the second dimension was performed in 1xTBE supplemented with 2 μg/ml chloroquine. The spot towards the top left corner corresponds to open circular DNA (OC), while the weaker spot diagonally below this corresponds to linear DNA (L).

$..GATC(G_3T_4)_3G_3GATC(G_3T_4)_4G_3GATC(G_3T_4)_4G_3GATCC_3A_4C_3A_4C_3GATC..$

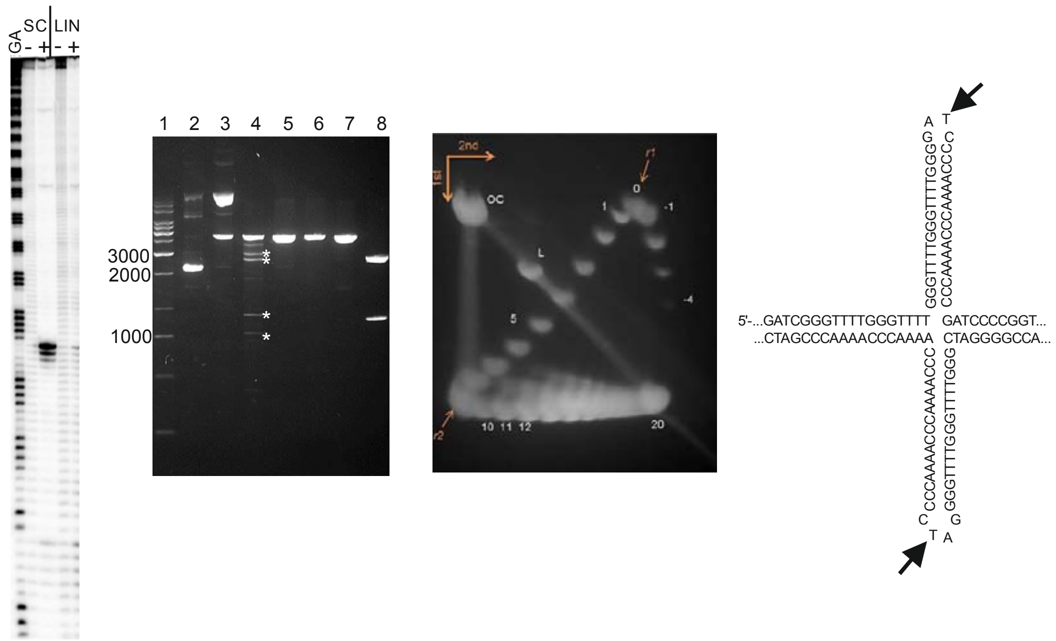

Figure S6. Properties of the plasmid containing three imperfect repeats of $(G_3T_4)_4$, including an inverted C_3A_4 repeat. First panel: reaction with potassium permanganate. - and + indicate reaction with permanganate. Second panel: S1 mapping: Lane 1, DNA marker ladder; lane 2, native supercoiled DNA; lane 3, cleavage with S1 nuclease; lane 4, digestion with S1 nuclease followed by ScaI; lane 5 digestion with ScaI; lane 6, digestion with *Sca*1 followed by S1 nuclease; lane 7, digestion with EcoRI; lane 8, digestion with EcoRI and ScaI. The products of S1 nuclease followed by ScaI digestion are indicated by the asterisks. Third panel: Two-dimensional electrophoresis of mixtures of plasmid topoisomers. The 1% agarose gel was run in 1xTBE buffer supplemented with 1 mM KCl. After running in the first dimension, the gel was placed in a dark container and soaked in TBE supplemented with 2 µg/ml chloroquine for about 7 hours. Electrophoresis in the second dimension was performed in 1xTBE supplemented with 2 µg/ml chloroquine. Fourth panel: Schematic showing the possible cruciform structure formed by the inverted repeat sequence in this plasmid. The arrows indicate the Ts that are hyperreactive to permanganate
